# Dynamic contrast-enhanced liver MRI in children: image quality comparison between GRASP-VIBE and CAIPIRINHA-VIBE

**DOI:** 10.1007/s00330-026-12635-z

**Published:** 2026-05-21

**Authors:** Simon Veldhoen, Anne Pohrt, Matthias Stephan Anders, Corona Metz

**Affiliations:** 1 Department of Radiology, Pediatric Radiology, Charité–Universitätsmedizin Berlin, corporate member of Freie Universität Berlin and Humboldt-Universität zu Berlin, Berlin, Germany; 2 Institute for Biometry and Clinical Epidemiology, Charité–Universitätsmedizin Berlin, corporate member of Freie Universität Berlin and Humboldt-Universität zu Berlin, Berlin Institute of Health, Berlin, Germany

**Keywords:** Pediatrics, Sequence comparison, Dynamic contrast-enhanced MRI, GRASP-VIBE, CAIPIRINHA-VIBE

## Abstract

**Objectives:**

To compare image quality between GRASP-VIBE (free-breathing) and CAIPIRINHA-VIBE (breath-hold) sequences in dynamic liver MRI in a pediatric population.

**Materials and methods:**

This retrospective study included 70 children who underwent dynamic liver MRI at 3 T with either GRASP-VIBE (*n* = 40) or CAIPIRINHA-VIBE (*n* = 40) acquisition. Ten patients underwent both imaging protocols at different time points. Image pairs were matched at four representative anatomical levels using vascular landmarks. Four dynamic contrast phases (pre-contrast, arterial, portal venous, delayed venous) at each anatomic level were independently reviewed by two radiologists, leading to a total of 640 comparisons per reader. Reader preference was assessed using a two-alternative forced-choice paradigm. Inter- and intra-reader agreement were analyzed using binomial tests and Cohen’s κ.

**Results:**

GRASP-VIBE was preferred over CAIPIRINHA-VIBE across all contrast-enhanced phases of pediatric liver MRI: 82.2% [526/640] in the arterial, 83.1% [532/640] in the portal venous, 71.3% [456/640] in the delayed venous (all *p* < 0.001). No significant difference was observed for the pre-contrast phase with 59.4% [380/640] GRASP-VIBE preference (*p* = 0.45). Strongest GRASP-VIBE preferences were observed in older children and adolescents and in examinations performed without anesthesia. Across all anatomical levels, GRASP consistently outperformed Caipirinha-VIBE in contrast-enhanced phases. Inter-reader agreement was moderate in the arterial (κ = 0.51) and delayed venous (κ = 0.48) phases, and slight in pre-contrast (κ = 0.19) and portal venous (κ = 0.20) phases (intra-reader agreement, κ = 0.56, κ = 0.60, κ = 0.28 and κ = 0.42).

**Conclusion:**

GRASP-VIBE offered superior subjective image quality compared to CAIPIRINHA-VIBE in pediatric liver MRI, especially in early contrast phases. Its free-breathing approach might be particularly advantageous regarding image quality in children unable to perform breath-holds.

**Key Points:**

***Question*** *Does free-breathing GRASP-VIBE provide superior image quality compared to breath-hold CAIPIRINHA-VIBE for dynamic liver MRI in pediatric patients?*

***Findings*** *GRASP-VIBE was preferred across all contrast phases, delivering consistently higher-quality images than CAIPIRINHA-VIBE, particularly in children unable to perform breath-holds.*

***Clinical relevance*** *Using GRASP-VIBE enables high-quality dynamic pediatric liver MRI. Further research is needed to determine whether this sequence can reduce the need for sedation or general anesthesia in children.*

**Graphical Abstract:**

**Figure:**
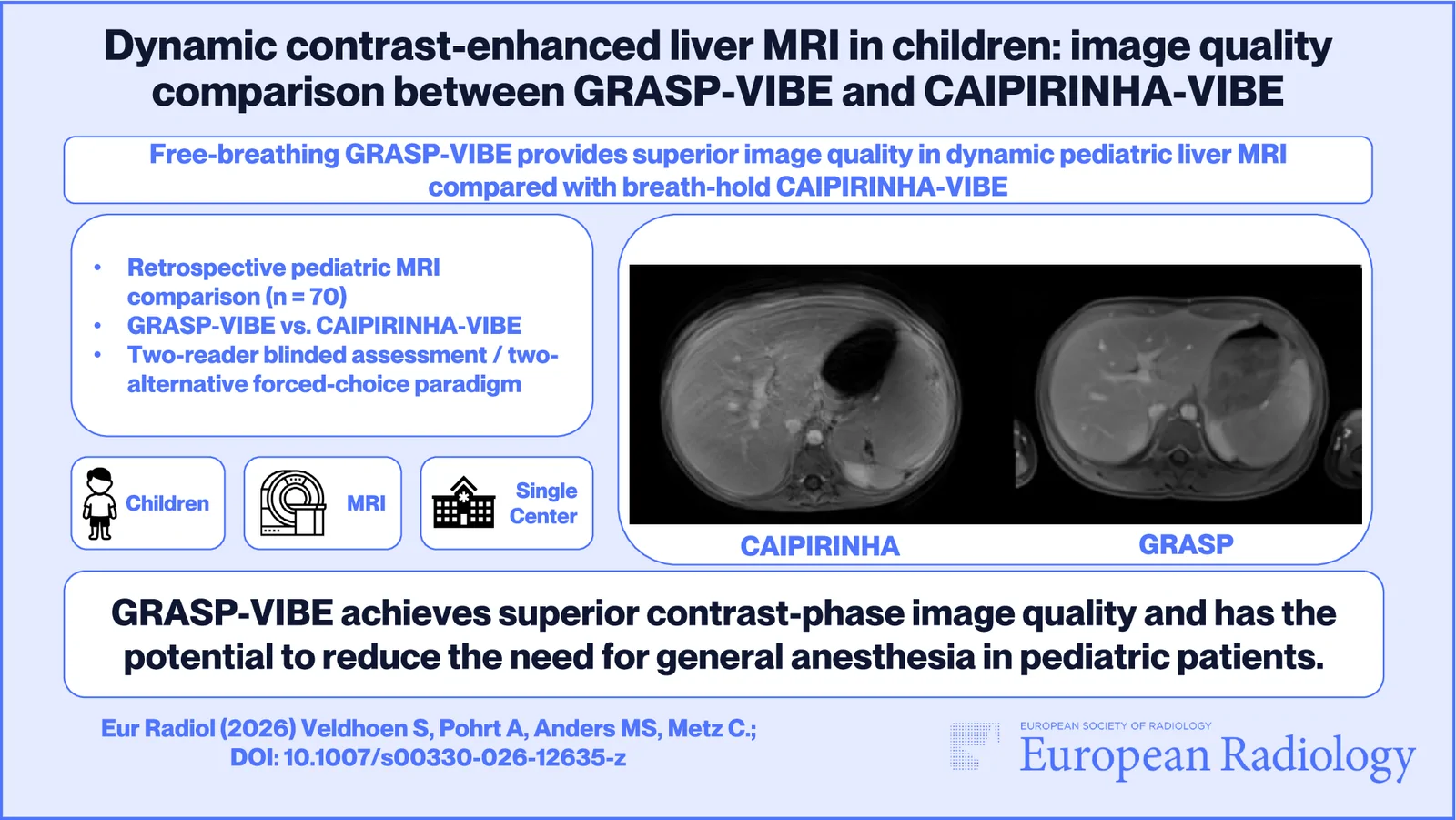

## Introduction

MRI of the liver is playing an increasingly important role in the diagnosis and monitoring of many diseases in children. It is the imaging modality of choice in evaluating focal liver lesions, vascular malformations, congenital anomalies, and oncologic follow-up, due to its superior soft-tissue contrast and ability to perform functional and perfusion imaging without radiation exposure [1–3].

A key element of diagnostic liver MRI is dynamic contrast-enhanced (DCE) imaging, typically acquired in multiple vascular phases (arterial, portal venous, and delayed venous). These dynamic series allow for accurate lesion detection and characterization, assessment of vascular structures, and evaluation of parenchymal perfusion [4, 5]. However, in pediatric patients, acquiring high-quality dynamic images is challenging due to a variable breath-hold capability, unpredictable cooperation, and faster circulation cycles compared to adults [6].

Traditional breath-hold T1-weighted 3D gradient echo sequences, such as CAIPIRINHA-VIBE (Controlled Aliasing in Parallel Imaging Results in Higher Acceleration Volume Interpolated Breathhold Examination), are widely used in clinical practice and are effective in cooperative adults and adolescents [7]. They rely on rapid acquisition during suspended respiration to minimize motion artifacts and improve temporal resolution. However, in young children, achieving adequate breath-holding is difficult, often resulting in non-diagnostic images [8, 9]. Therefore, DCE liver MRI in children often requires the administration of anesthesia or sedation, particularly when sequences rely on breath-hold performance.

To address these challenges, free-breathing acquisition techniques have been developed. One such method is GRASP-VIBE (Golden-angle Radial Sparse Parallel), which combines radial k-space sampling, compressed sensing, and parallel imaging to allow continuous acquisition of dynamic data during free breathing [10]. GRASP-VIBE enables retrospective reconstruction of multiple contrast phases with intrinsic motion resistance and flexible temporal resolution. This makes it especially attractive for use in pediatric imaging, where voluntary breath-hold compliance is limited [6].

Although feasibility studies have shown that GRASP-VIBE can provide diagnostically useful dynamic liver imaging in adults [10–12], there are no direct comparisons to standard sequences such as CAIPIRINHA-VIBE and data on children. Therefore, the aim of this study was to evaluate and compare the image quality of GRASP-VIBE and CAIPIRINHA-VIBE in pediatric DCE liver MRI.

## Materials and methods

### Institutional review

The study was approved by the institutional review board (Charité–Universitätsmedizin Berlin).

### Study design and population

This retrospective study included pediatric patients who underwent liver MRI in a 2-year period at a single tertiary center between September 2022 and June 2024. Inclusion criteria comprised the availability of a complete GRASP- or CAIPIRINHA-VIBE sequence across the dynamic contrast-enhanced phases and a patient age below 18 years at the time of imaging. The sole exclusion criterion was the absence of the corresponding sequence within the imaging dataset. The use of GRASP-VIBE and CAIPIRINHA-VIBE sequences reflected a temporal modification of the institutional imaging protocol, with CAIPIRINHA-VIBE representing the initial standard technique and GRASP-VIBE being introduced subsequently as part of a protocol update. Patients underwent the respective sequence depending on the period in which their examination was performed. To ensure comparability between groups, cases from the available pool of eligible examinations were selected to achieve similar age distributions between cohorts. For each GRASP-VIBE case, patients of comparable age (± 1 year) from the available CAIPIRINHA-VIBE datasets were selected.

### MRI protocol and acquisition

All examinations were performed on the same 3-T MRI system (Magnetom Skyra, Siemens Healthineers) using standard pediatric liver MRI protocols and identical software during the course of the study. The GRASP-VIBE sequence was acquired during free breathing. The CAIPIRINHA-VIBE sequence was obtained during breath-hold. The sequence parameters of both sequences are summarized in Table 1. Contrast agent (0.1 mmol/kg) was administered intravenously at 1 mL/s with a 20 mL saline flush. CAIPIRINHA-VIBE imaging included four phases: Pre-contrast, arterial phase (approx. 20–30 s post-injection), portal venous phase (60–70 s), delayed venous phase (~ 180 s).

**Table 1:** Sequence parameter of GRASP-VIBE and CAIPIRINHA-VIBE

Parameter	Unit	GRASP-VIBE	CAIPIRINHA-VIBE
Total acquisition time	s	313 (total)	14 (per phase)
Echo time	ms	3.74–4	4.3
Time of echo	ms	1.59–1.96	2.1
Field of view	mm × mm	200 × 200–300 × 300	260 × 320
In-plane resolution	mm × mm	1.05 × 1.05–1.34 × 1.34	1.00 × 1.00
Slice thickness	mm	3	3
Number of slices	#	50–114	68–78
Bandwidth	Hz/Px	457–587	401
Flip angle	°	8.5–12	9
Fat saturation	Yes/no	Yes	Yes
Trajectory		Stack-of-Stars	Cartesian
Acceleration		Compressed sensing with golden-angle radial sampling	Parallel imaging with CAIPIRINHA (factor 4)
Temporal resolution		Continuous acquisition, reconstructed into 6–10 s phases	Fixed acquisition, reconstructed at 15, 50, 90, 120 s
Breathing state		Free breathing with liver gating	Breath-hold (14 s)

*GRASP* golden-angle radial sparse parallel, *CAIPIRINHA* controlled aliasing in parallel imaging

Since GRASP-VIBE data is acquired continuously during free breathing, unlimited time points of the continuous acquisition can be reconstructed. Representative images corresponding to the four time phases of conventional CAIPIRINHA-VIBE phase timing were manually selected from the GRASP-VIBE dataset by one of the readers, who chose the images best representing each phase.

### Image assessment

Two radiologists fellowship-trained in pediatric radiology independently (13 and 7 years of experience) reviewed the original anonymized DICOM image files on a clinical PACS workstation using a dedicated comparison platform [13]. One of the readers selected representative images for each anatomical level prior to evaluation. All images could be windowed and leveled within the platform to optimize visualization. A two-alternative forced-choice paradigm was used to assess subjective image quality [13–16]. Readers were presented with paired images from GRASP- and CAIPIRINHA-VIBE acquisitions from the same contrast phase and asked to indicate which image provided better diagnostic quality based on motion artifacts, image sharpness, contrast timing, vessel and organ definition, and overall diagnostic confidence. Overall diagnostic confidence was assessed subjectively by the two pediatric radiologists for each image pair, with readers indicating which sequence provided greater confidence in establishing a reliable diagnosis. This structured evaluation reflects standard clinical decision-making.

For each phase, image pairs were matched at four representative anatomical levels using vascular landmarks to assess the influence of anatomic location on readers’ preferences: the hepatocaval confluence of the hepatic vein, the peripheral hepatic veins between the hepatocaval confluence and the hepatic hilum, the hepatic hilum and the origin of the celiac trunk. Given the sample size of 40 images per group, this resulted in 40 pairwise comparisons per anatomical level, yielding 160 comparisons per phase and a total of 640 comparisons across all four contrast phases per reader. The patient pairs were age-matched to reduce potential age-related bias in image quality assessment.

A subanalysis was performed to assess the influence of anesthesia on readers’ preferences. Of the 640 image pairs, 366 were from examinations performed without anesthesia, 240 were from examinations performed with anesthesia, and 34 comparisons were mixed and therefore excluded from this subanalysis.

An additional subanalysis was performed to assess the influence of patient age on reader preferences. Age groups were defined according to typical clinical practice, with children up to 6 years (in whom MRI scans are usually performed under anesthesia), children aged 6–12 years (typically scanned without anesthesia), and adolescents older than 12 years analyzed as a separate third group.

One reader conducted the image assessment twice, with an interval of 3 months in between to measure the intra-rater agreement of the scoring.

**Fig. 1:**
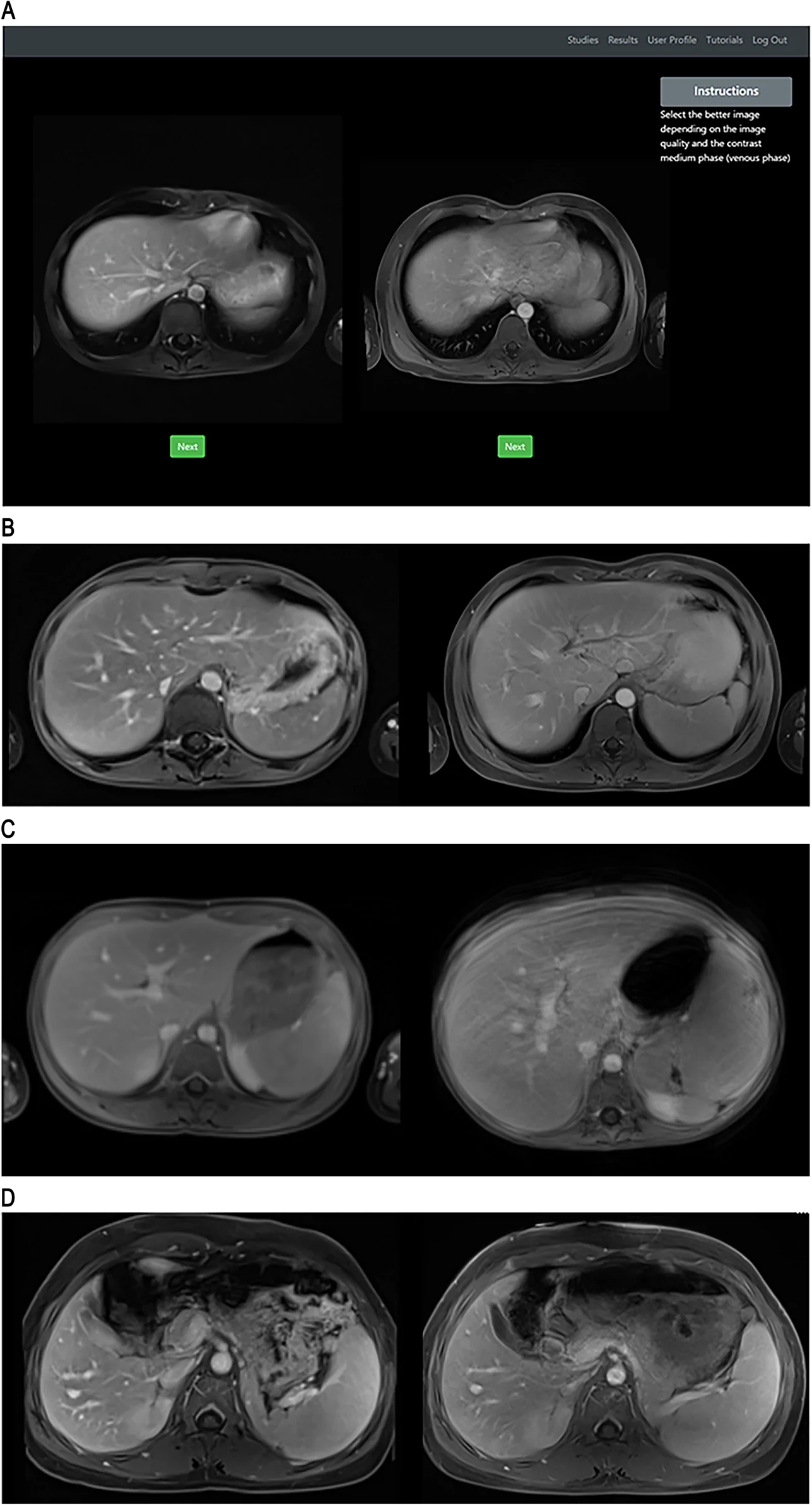
Examples of paired image comparisons (left side CAIPIRINHA-VIBE; right side GRASP-VIBE) at the anatomical levels assessed during the venous phase. **A** Full screenshot from the software used for image comparison at the level of the hepatocaval confluence. Readers had to choose image preference by clicking one of the “Next” buttons below the respective image. Both readers preferred CAPIRINHA-VIBE. **B** Image pair from the level of the periphal hepatic veins. Both readers preferred GRASP-VIBE. **C** Image pair from the level of the hepatic hilum. Both readers preferred GRASP-VIBE. **D** Image pair from the level of the celiac trunk. Both readers preferred CAIPIRINHA-VIBE

An example of the paired image comparison interface used in the assessment is shown in Fig. 1.

### Statistical analysis

Descriptive statistics were used to summarize patient demographics and imaging parameters. For each phase and (sub-) group, reader preferences were analyzed using a mixed effects model and tested against a 50% null hypothesis. Intra- and inter-reader agreement were evaluated using Cohen’s kappa with the categories no, slight, fair, moderate, substantial and near perfect agreement [17] and agreement rates (proportion of image pairs in which both readers selected the same imaging protocol as their preferred sequence in percentage). Differences between GRASP preferences in cases with anesthesia and without, as well as differences in GRASP preferences between patient age groups, were analyzed using mixed effects models with random effects for reader and comparison. A *p*-value < 0.05 was considered statistically significant.

## Results

### Study population

A total of 70 pediatric patients were included in the study. The GRASP-VIBE cohort (*n* = 40) had a mean age of 8.5 years (median 8, range 0–17) with 19 females, while the CAIPIRINHA-VIBE cohort (*n* = 40) had a mean age of 8.4 years (median 8, range 0–17) with 18 females. Ten patients underwent both imaging procedures on different days as part of their clinical follow-up. One CAIPIRINHA and two GRASP-VIBE patients received gadoxetic acid (Primovist®, Bayer); all other patients received gadobutrol (Gadovist®, Bayer), each administered at the clinical standard dose of 0.1 mL/kg. The indications for MRI were grouped into five categories: oncologic follow-up, congenital liver disease, infection/inflammation, incidental lesions, and vascular anomalies. Distribution across indications was balanced between groups (Table 2).

**Table 2:** Categorization of the study cohorts

	GRASP-VIBE	CAIPIRINHA-VIBE	*p*-value
Sex (female/male), *n*	19/21	18/22	1
Age (years), mean (sd)	8.5 (5.4)	8.4 (5.6)	0.95
Sedation, *n* (%)	16 (40%)	16 (40%)	1
Indication category			
Tumor follow-up/oncological diseases	17	19	
Pancreatic diseases	4	1	
Liver and biliary tract diseases	4	9	
Congenital/genetic disorders	4	4	
Other inflammatory/autoimmune/vascular conditions	4	4	
Total	40	40	

*GRASP* golden-angle radial sparse parallel, *CAIPIRINHA* controlled aliasing in parallel imaging results in higher acceleration, *VIBE* volumetric interpolated breath-hold examination, *sd* standard deviation

### Overall preference

**Fig. 2:**
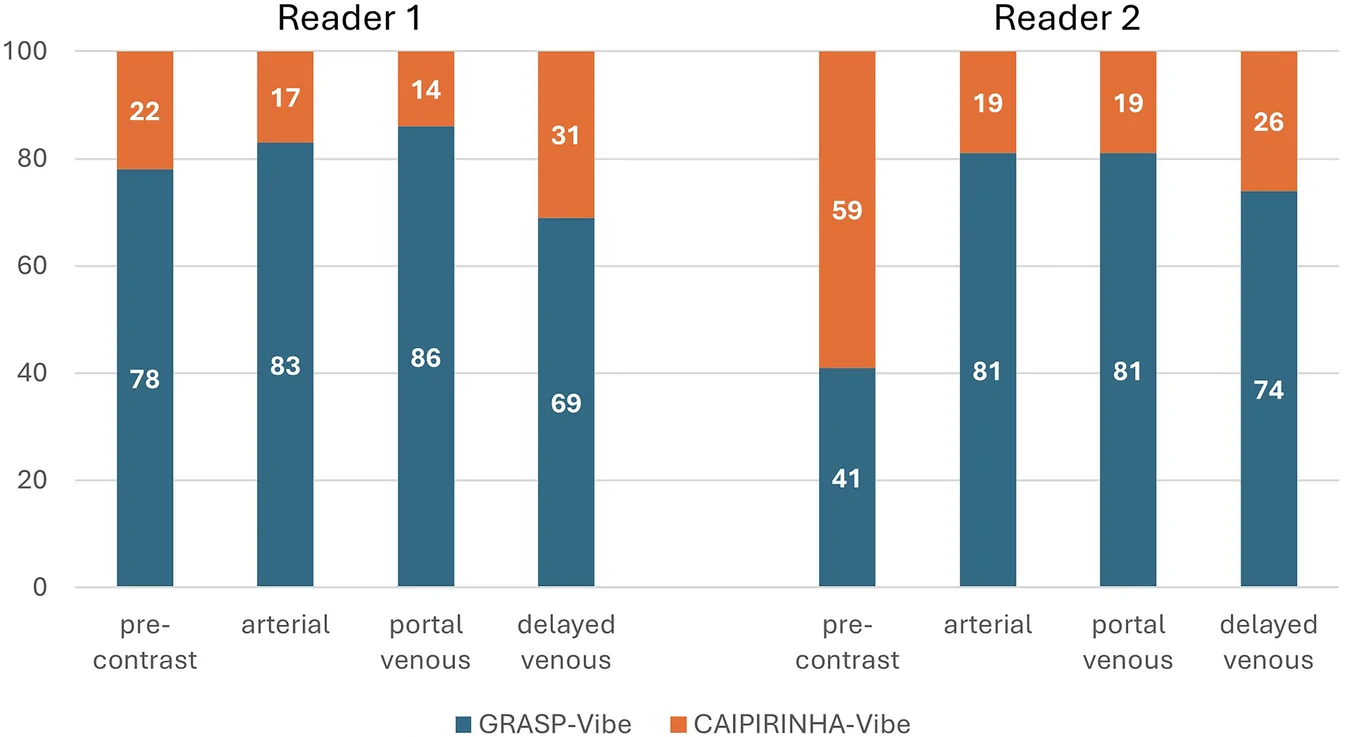
Distribution of image ratings for each reader and each contrast phase

Across all evaluated post-contrast phases, GRASP-VIBE was preferred over CAIPIRINHA-VIBE. Preference was most pronounced in the portal venous phase, with GRASP-VIBE being selected in 83.1% (*p* < 0.001), followed closely by the arterial phase (82.2%, *p* < 0.001). In the delayed venous phase, GRASP-VIBE was favored in 71.3% of cases (*p* < 0.001). In the pre-contrast phase, GRASP-VIBE was chosen in 59.4% (*p* = 0.45). This lower value was mainly driven by a strong preference of the more experienced reader for CAIPIRINHA-VIBE. Figure 2 illustrates the distribution of ratings per reader and phase; a detailed breakdown of the results, including all sub-analyses, is provided in Table 3.

**Table 3:** Preference for the GRASP-VIBE sequence vs. CAIPIRINHA-VIBE according to contrast phase, anesthesia, anatomical region and age group—percentage, statistical significance and inter/intra-reader agreement

	Pre-contrast	Arterial	Portal venous	Delayed venous	Total
Overall, *n* = 320					
GRASP preference (%)	59.4	82.2	83.1	71.3	74.0
*p*-value	0.45	< 0.0001	< 0.0001	< 0.001	< 0.0001
κ (inter/intra)	0.19/0.28	0.51/0.56	0.20/0.42	0.48/0.6	0.34/0.48
Anesthesia					
With anesthesia, *n* = 120					
GRASP preference (%)	52.5	78.3	81.7	64.2	69.2
*p*-value	0.83	< 0.0001	< 0.0001	0.17	< 0.0001
κ (inter/intra)	0.08/0.34	0.51/0.47	0.23/0.52	0.42/0.54	0.30/0.50
Without anesthesia, *n* = 184					
GRASP preference (%)	65.8	86.4	85.6	78.8	79.1
* p*-value	0.23/n.s	< 0.0001	< 0.0001	< 0.0001	< 0.0001
κ (inter/intra)	0.23/0.16	0.49/0.58	0.10/0.32	0.45/0.49	0.31/0.38
With anesthesia vs. without anesthesia (odds ratio)	0.46	0.5	0.72	0.26	0.60
* p*-value	0.02	0.52	0.42	0.02	< 0.001
Anatomy					
Hepatocaval confluence, *n* = 80					
GRASP preference (%)	52.5	85.0	81.3	76.3	73.8
*p*-value	0.83	< 0.0001	< 0.0001	< 0.0001	< 0.0001
κ (inter/intra)	0.20/0.46	0.42/0.55	−0.04/0.22	0.53/0.61	0.30/0.50
Peripheral liver veins, *n* = 80					
GRASP preference (%)	60.0	84.2	85.0	66.3	73.9
* p*-value	0.52	< 0.0001	0.03	0.23	0.0001
κ (inter/intra)	0.01/0.03	0.54/0.59	0.23/0.44	0.61/0.57	0.31/0.41
Liver hilus, *n* = 80					
GRASP preference (%)	61.3	81.3	85.0	75.0	75.6
* p*-value	0.38	< 0.0001	< 0.0001	< 0.0001	< 0.0001
κ (inter/intra)	0.38/0.30	0.75/0.66	0.42/0.48	0.34/0.48	
Celiac trunk, *n* = 80					
GRASP preference (%)	63.8	78.2	81.3	67.5	72.6
* p*-value	0.25	0.01	0.01	0.03	< 0.0001
κ (inter/intra)	0.18/0.35	0.32/0.42	0.26/0.48	0.43/0.72	0.29/0.50
Age					
0–5 years, *n* = 120					
GRASP preference (%)	51.7	68.3	83.3	57.5	65.2
*p*-value	0.90	< 0.0001	< 0.0001	0.42	< 0.001
κ (inter/intra)	−0.01/0.17	0.62/0.60	0.19/0.34	0.46/0.56	0.30/0.48
6–12 years, *n* = 96					
GRASP preference (%)	64.6	87.2	79.2	78.1	77.2
* p*-value	0.17	< 0.0001	< 0.0001	< 0.0001	< 0.001
κ (inter/intra)	0.40/0.65	0.43/0.60	0.26/0.73	0.43/0.67	0.43/0.67
> 12 years, *n* = 96					
GRASP preference (%)	63.5	92.9	85.2	80.2	80.4
* p*-value	0.32	< 0.0001	< 0.0001	< 0.0001	< 0.001
κ (inter/intra)	0.27/0.16	−0.04/−0.04	0.21/0.40	0.42/0.40	0.28/0.29

*n*: number of cases, κ: Cohen’s kappa (first number is inter-reader-agreement)

### Influence of anesthesia

The use of anesthesia markedly influenced the preference patterns:

- Without anesthesia, preference for GRASP-VIBE was consistently higher across all phases (arterial 86.4%, portal venous 85.6%, delayed venous 78.8%, all *p* < 0.001) compared to with anesthesia (arterial 78.3%, *p* < 0.001; portal venous 81.7%, *p* < 0.001; delayed venous 64.2%, *p* = 0.17).
- In the pre-contrast phase, GRASP-VIBE preference without anesthesia reached 65.8% (*p* = 0.32), whereas with anesthesia the preference dropped to 52.5% (*p* = 0.83).
- Preference for GRASP was significantly higher without anesthesia compared to with anesthesia in the pre-contrast phase (*p* = 0.02) and the venous phase (*p* = 0.02), and in total (*p* < 0.001).

### Influence of the anatomical level

The anatomical level of image comparison had no significant impact on the dominant trend toward GRASP-VIBE preference in contrast-enhanced phases. From the arterial phase onward, GRASP-VIBE was consistently rated superior across anatomical levels. Some variations emerged:

- Arterial phase preferences were high across all anatomical levels (78.2–85%, all *p* < 0.01).
- Portal venous phase preferences remained high throughout all anatomical levels (81.3–85.0%, all *p* < 0.05).
- Pre-contrast phases across all anatomical levels showed markedly lower preferences for GRASP-VIBE (52.5–63.8%), which were not statistically significant.

### Age dependency

Preference for GRASP-VIBE increased with patient age, peaking in adolescents.

- 0–5 years (*n* = 120): lowest GRASP-VIBE preference across phases (arterial 68.3%, *p* < 0.001, delayed venous 57.5%, *p* = 0.43, n.s). Preference in the pre-contrast phase was 51.7%, *p* = 0.90—the lowest among age groups.
- 6–12 years (*n* = 96): markedly higher values (arterial 87.2%, *p* < 0.001, delayed venous 78.1%, *p* < 0.001).
- > 12 years (*n* = 96): highest preference rates, especially arterial (92.9%, *p* < 0.0001), delayed venous (80.2%, *p* < 0.001), and portal venous (85.2%, *p* < 0.001).
- In the delayed venous phase, GRASP preference was significantly higher in the older age groups than in the younger age group.

### Inter-reader agreement

The overall inter-reader agreement across all phases was fair, with κ = 0.34 or 74% agreement rate. Agreement varied between the contrast phases analyzed: Cohen’s kappa showed moderate reliability in the arterial phase (κ = 0.51, 86% agreement) and the delayed venous phase (κ = 0.48, 79% agreement) and fair agreement in the portal venous phases (κ = 0.21, 78% agreement), whereas agreement was low in the pre-contrast (κ = 0.18, 55% agreement). Detailed results of inter-reader agreement for each phase are summarized in Table 3.

### Intra-reader agreement

The overall intra-reader agreement across all phases was moderate, with κ = 0.60 or 80% agreement rate. Agreement varied between phases: Cohen’s kappa showed substantial reliability in the arterial phase (κ = 0.71, 86% agreement), delayed venous phase (κ = 0.64, 82% agreement) and portal venous phases (κ = 0.67, 84% agreement), whereas agreement was fair in the pre-contrast phase (κ = 0.38, 69% agreement). Detailed results of intra-reader agreement for each phase are summarized in Table 3.

## Discussion

The two readers significantly preferred GRASP-VIBE over CAIPIRINHA-VIBE images across all contrast-enhanced phases of pediatric liver MRI. The results were consistent across readers and especially strong in the arterial and portal venous contrast phases, where GRASP-VIBE was preferred in up to 83% of overall cases.

Retrospectively reconstructed GRASP-VIBE provided consistent, high-quality arterial phase images throughout all patients. Although it is known that transient motion artifacts in arterial phase breath-hold imaging are less frequent in children [8, 18], the free-breathing nature of GRASP-VIBE, combined with its retrospective reconstruction capability, reduces dependence on bolus tracking and breath-hold precision. This is of value for pediatric imaging, especially in the presence of poor compliance, as strongly reflected by the increased preference for GRASP-VIBE in procedures performed without general anesthesia. Across all phases, preference rates for GRASP-VIBE were consistently higher in the non-anesthesia subgroup (for example, 86% in arterial and 86% in portal venous phases), underscoring the robustness of this approach in routine, non-sedated pediatric practice. Subgroup analyses further showed the influence of age and sedation status on sequence preference. Younger children (0–5 years) and those who underwent MRI under anesthesia (largely overlapping populations) showed the lowest, albeit still positive, preference for GRASP-VIBE. In contrast, older children and adolescents, who were predominantly examined without anesthesia, showed significantly higher preference rates. This pattern likely reflects the different breathing conditions during image acquisition. Under general anesthesia with controlled mechanical ventilation, respiratory motion can be effectively managed and breath-hold commands can be reliably implemented. In this setting, image quality differences between GRASP-VIBE and CAIPIRINHA-VIBE are less pronounced. Conversely, in non-sedated children examined under free-breathing conditions, breath-hold compliance may be variable in clinical routine. Under these circumstances, the motion robustness of GRASP-VIBE becomes more relevant, thereby increasing its relative advantage. In the portal venous and delayed phases, readers preferred GRASP-VIBE in over 70% of cases. This advantage was robust across all anatomical regions and particularly evident in non-sedated and older pediatric patients.

Overall inter-reader agreement was moderate (κ = 0.34), reflecting agreement at the level of individual image ratings. The relatively low inter-reader agreement observed in some phases likely reflects subtle differences in image quality and the binary forced-choice design, which requires a preference even in near-equivalent image pairs. This may increase variability at the individual comparison level. Importantly, both readers independently demonstrated a consistent overall preference for GRASP-VIBE across all contrast-enhanced phases, suggesting that the observed effect is systematic rather than random. Among the evaluated phases, the arterial phase showed the numerically highest agreement. Lower κ-values in the pre-contrast and portal venous phases likely reflect the subtler image differences present in these settings or more subjective thresholds for artifact tolerance.

From a clinical perspective, GRASP-VIBE may enable high-quality liver imaging in a larger proportion of pediatric patients. By mitigating the need for cooperation and breath-holding, it may reduce the need for general anesthesia, procedure time, patient risk, and institutional costs. While GRASP-VIBE enables flexible reconstruction of multiple contrast phases, this may result in increased data volume and potentially higher workflow demands. However, in clinical practice, reconstruction can be limited to a small number of diagnostically relevant phases, thereby mitigating this effect.

### Study limitations

The study limitations include the retrospective design and qualitative nature of image assessment. Image quality assessment relied solely on structured subjective evaluation by experienced pediatric radiologists without objective or semi-quantitative metrics such as signal-to-noise or contrast-to-noise ratios. While this reflects clinical decision-making in routine practice, it limits quantitative comparison between sequences. The cohort size, while sufficient for initial comparisons, may not reflect rare pathologies or broader age distributions. Due to ethical and practical constraints in pediatric imaging, intra-individual comparisons between GRASP- and CAIPIRINHA-VIBE sequences were not feasible. Except for a single patient—who underwent both sequences within a short time interval for clinically indicated reasons—all comparisons were performed between different individuals. For the other nine patients who received both imaging protocols, the interval between examinations was too long to allow meaningful intra-individual comparisons while maintaining appropriate age comparability. Additionally, only three patients received gadoxetic acid, limiting contrast-specific subgroup analysis. Motion artifacts can lead to “steps” between axial layers that are not recognizable on individual layers. This could have contributed to underestimating the overall benefit of free-breathing GRASP in this study design. No correlation with clinical outcome or lesion detection accuracy was performed. A reliable evaluation of lesion detection would require a substantially different study design, and such an investigation would be valuable and is, in our view, an important future research direction. Intra-reader agreement was calculated based on two repeated ratings performed by a single reader only. A potential limitation of this study is that representative images for GRASP-VIBE phases were manually selected by one of the readers to best represent each standard contrast phase, whereas the corresponding phases in CAIPIRINHA-VIBE are automatically provided by the acquisition protocol. Of course, contrast timing is not an intrinsic property of the imaging sequence; the ability to obtain optimally timed phases, however, is an important aspect of clinical image quality. While this manual selection could introduce a source of bias in subjective image quality assessment, it also highlights a key clinical advantage of GRASP-VIBE, allowing optimal phase identification even in patients with irregular breathing or variable contrast timing. Despite these limitations, the study provides robust evidence in support of GRASP-VIBE for routine pediatric liver imaging. Further prospective studies should assess diagnostic accuracy, contrast agent-specific effects, and long-term clinical utility.

## Conclusion

This study demonstrates that GRASP-VIBE significantly outperforms CAIPIRINHA-VIBE in terms of reader preference across all post-contrast phases of pediatric liver MRI. The superiority of GRASP-VIBE was particularly evident in the arterial and portal venous phases. GRASP-VIBE’s free-breathing approach and flexible retrospective reconstruction make it a robust and patient-friendly alternative and may reduce the need for general anesthesia, as it does not require breath-hold maneuvers, enhancing workflow efficiency. As motion-robust techniques become more widely available, they are likely to become standard-of-care in pediatric abdominal imaging. Future research should explore broader clinical applications, including lesion detection performance and quantitative perfusion analysis.

## Supplementary information

ELECTRONIC SUPPLEMENTARY MATERIAL

## Abbreviations

CAIPIRINHA-VIBE: Controlled aliasing in parallel imaging results in higher acceleration
DCE: Dynamic contrast-enhanced
GRASP-VIBE: Golden-angle radial sparse parallel
VIBE: Volume interpolated breathhold examination
