# Supplementary material for: Dynamic contrast-enhanced liver MRI in children: image quality comparison between GRASP-VIBE and CAIPIRINHA-VIBE
Source: Eur Radiol. 2026 May 21;36(10):7796–805. doi: 10.1007/s00330-026-12635-z (PMC13574926; doi:10.1007/s00330-026-12635-z)
Supplement: Supplementary file 1 [file 330_2026_12635_MOESM1_ESM.pdf]

Dynamic contrast-enhanced liver MRI in children: image quality comparison between GRASP-VIBE and CAIPIRINHA-VIBE

ELECTRONIC SUPPLEMENTARY MATERIAL

Eur Radiol (2026) Veldhoen S, Pohrt A, Anders MS, Metz C.

Supplemental table 1a: GRASP preference differences between groups

GRASP preference in each group is compared to GRASP preference in each other group using mixed models. Odds ratios are reported together with 95%-CIs and p-values.

GRASP Preference differences between Anesthesia groups

		With anesthesia	Without anesthesia
With anesthesia vs.	OR		0.597
	95%-CI		[-0.79; -0.241]
	p		<0.001
Without anesthesia vs	OR	1.674	
	95%-CI	[0.241; 0.79]	
	p	<0.001	

Anatomy: GRASP Preference differences between heights

		hilus	coeliac trunk	hepatocaval confluence	Peripheral liver veins
hilus vs.	OR		1.27	1.119	1.146
	95%-CI		[-0.142; 0.62]	[-0.271; 0.496]	[-0.244; 0.518]
	p		0.218	0.565	0.482
coeliac trunk vs	OR	0.787		0.881	0.902
	95%-CI	[-0.62; 0.142]		[-0.506; 0.253]	[-0.48; 0.274]
	p	0.218		0.513	0.593
hepatocaval confluence	OR	0.894	1.135		1.024
	95%-CI	[-0.496; 0.271]	[-0.253; 0.506]		[-0.351; 0.399]
	p	0.5645	0.513		0.9
Peripheral liver veins vs.	OR	0.872	1.108	0.976	
	95%-CI	[-0.518; 0.244]	[-0.274; 0.48]	[-0.399; 0.351]	
	p	0.482	0.593	0.9	

GRASP Preference differences between age groups

	comparison	0-5y	6-12y	>12y
0-5y vs.	OR		0.558	0.45
	95%-CI		[-0.911; -0.257]	[-1.128; -0.469]
	p		<0.001	<0.001
6-12y vs	OR	1.792		0.807
	95%-CI	[0.257; 0.91]		[-0.582; 0.153]
	p	<0.001		0.253
>12y vs	OR	2.209	1.239	
	95%-CI	[0.463; 1.122]	[-0.153; 0.582]	
	p	<0.001	0.253	

Supplemental table 1b: GRASP preference differences between phases

GRASP preference in each phase is compared to GRASP preference in each other phase using mixed models. Odds ratios are reported together with 95%-CIs and p-values.

Overall: GRASP Preference differences between Phases					
		pre-contrast	arterial	portal venous	delayed venous
Nativ vs.	OR		0.285	0.265	0.558
	95%-CI		[-1.635; -0.878]	[-1.71; -0.943]	[-0.928; -0.239]
	p		<0.001	<0.001	<0.001
arterial vs	OR	3.514		0.933	1.960
	95%-CI	[0.878; 1.635]		[-0.487; 0.347]	[0.287; 1.059]
	p	<0.001		0.742	<0.001
Portal venous vs	OR	3.768	1.072		2.102
	95%-CI	[0.943; 1.71]	[-0.347; 0.486]		[0.353; 1.133]
	p	<0.001	0.742		<0.001
delayed venous	OR	1.793	0.510	0.476	
	95%-CI	[0.239; 0.928]	[-1.059; -0.287]	[-1.133; -0.353]	
	p	<0.001	<0.001	<0.001	

With anesthesia: GRASP Preference differences between Phases					
		pre-contrast	arterial	portal venous	delayed venous
pre-contrast	OR		0.282	0.239	0.604
	95%-CI		[-1.893; -0.639]	[-2.068; -0.794]	[-1.083; 0.075]
	p		<0.001	<0.001	0.088
arterial vs	OR	3.546		0.848	2.142
	95%-CI	[0.639; 1.893]		[-0.862; 0.532]	[0.155; 1.369]
	p	<0.001		0.642	0.014
Portal venous vs	OR	4.184	1.180		2.527
	95%-CI	[0.794; 2.068]	[-0.532; 0.862]		[0.264; 1.59]
	p	<0.001	0.642		0.006
delayed venous	OR	1.655	0.467	0.396	
	95%-CI	[-0.075; 1.083]	[-1.369; -0.155]	[-1.59; -0.264]	
	p	0.088	0.014	0.006	

Without anesthesia: GRASP Preference differences between Phases					
		pre-contrast	arterial	portal venous	delayed venous
pre-contrast	OR		0.250	0.275	0.450
	95%-CI		[-1.965; -0.81]	[-1.854; -0.728]	[-1.313; -0.285]
	p		<0.001	<0.001	0.002
arterial vs	OR	4.004		1.101	1.802
	95%-CI	[0.81; 1.965]		[-0.518; 0.71]	[0.025; 1.153]
	p	<0.001		0.758	0.041
Portal venous vs	OR	3.636	0.908		1.636
	95%-CI	[0.728; 1.854]	[-0.71; 0.518]		[-0.08; 1.065]

	p	<0.001	0.758		0.092
delayed venous	OR	2.223	0.555	0.611	
	95%-CI	[0.285; 1.313]	[-1.153; -0.025]	[-1.065; 0.08]	
	p	0.002	0.041	0.092	

Height Hilus: GRASP Preference differences between Phases

		pre-contrast	arterial	portal venous	delayed venous
pre-contrast	OR		0.361	0.270	0.525
	95%-CI		[-1.94; -0.098]	[-2.282; -0.339]	[-1.523; 0.233]
	p		0.030	0.009	0.150
arterial vs	OR	2.770		0.747	1.453
	95%-CI	[0.098; 1.94]		[-1.324; 0.74]	[-0.581; 1.329]
	p	0.030		0.579	0.443
Portal venous vs	OR	3.709	1.339		1.946
	95%-CI	[0.339; 2.282]	[-0.74; 1.324]		[-0.31; 1.641]
	p	0.009	0.579		0.181
delayed venous	OR	1.906	0.688	0.514	
	95%-CI	[-0.233; 1.523]	[-1.329; 0.581]	[-1.641; 0.31]	
	p	0.150	0.443	0.181	

Height Truncus: GRASP Preference differences between Phases

		pre-contrast	arterial	portal venous	delayed venous
pre-contrast	OR		0.432	0.308	0.793
	95%-CI		[-1.727; 0.049]	[-2.106; -0.248]	[-1.069; 0.606]
	p		0.064	0.013	0.587
arterial vs	OR	2.315		0.713	1.836
	95%-CI	[-0.049; 1.727]		[-1.293; 0.618]	[-0.288; 1.503]
	p	0.064		0.488	0.184
Portal venous vs	OR	3.245	1.402		2.574
	95%-CI	[0.248; 2.106]	[-0.618; 1.293]		[0.01; 1.881]
	p	0.013	0.488		0.048
delayed venous	OR	1.261	0.545	0.389	
	95%-CI	[-0.606; 1.069]	[-1.503; 0.288]	[-1.881; -0.01]	
	p	0.587	0.184	0.048	

Height LVS1: GRASP Preference differences between Phases

		pre-contrast	arterial	portal venous	delayed venous
pre-contrast	OR		0.145	0.212	0.290
	95%-CI		[-2.848; -1.012]	[-2.389; -0.711]	[-2.044; -0.432]
	p		<0.001	<0.001	0.003
arterial vs	OR	6.891		1.463	1.998
	95%-CI	[1.012; 2.848]		[-0.537; 1.298]	[-0.199; 1.584]
	p	<0.001		0.416	0.128
Portal venous vs	OR	4.710	0.684		1.366
	95%-CI	[0.71; 2.389]	[-1.298; 0.537]		[-0.53; 1.154]
	p	<0.001	0.416		0.468
delayed venous	OR	3.448	0.500	0.732	
	95%-CI	[0.432; 2.044]	[-1.584; 0.199]	[-1.154; 0.53]	

		pre-contrast	arteriell	portal venous	delayed venous
	p	0.003	0.128	0.468	

Height LVS2: GRASP Preference differences between Phases

		pre-contrast	arteriell	portal venous	delayed venous
pre-contrast	OR		0.238	0.226	0.747
	95%-CI		[-2.3; -0.573]	[-2.357; -0.616]	[-1.036; 0.451]
	p		0.001	<0.001	0.441
Arteriell vs	OR	4.207		0.952	3.141
	95%-CI	[0.573; 2.3]		[-0.985; 0.886]	[0.28; 2.009]
	p	0.001		0.917	0.009
Portal venous vs	OR	4.421	1.051		3.301
	95%-CI	[0.616; 2.357]	[-0.886; 0.985]		[0.327; 2.062]
	p	<0.001	0.917		0.007
delayed venous	OR	1.340	0.318	0.303	
	95%-CI	[-0.451; 1.036]	[-2.009; -0.28]	[-2.062; -0.327]	
	p	0.441	0.009	0.007	

Age 0-5y: GRASP Preference differences between Phases

		pre-contrast	arterial	portal venous	delayed venous
pre-contrast	OR		0.550	0.222	0.883
	95%-CI		[-1.188; -0.009]	[-2.146; -0.867]	[-0.699; 0.451]
	p		0.047	0.000	0.672
arterial vs	OR	1.819		0.403	1.606
	95%-CI	[0.009; 1.188]		[-1.588; -0.229]	[-0.085; 1.033]
	p	0.047		0.009	0.096
Portal venous vs	OR	4.511	2.480		3.984
	95%-CI	[0.867; 2.146]	[0.229; 1.588]		[0.711; 2.053]
	p	0.000	0.009		<0.001
delayed venous	OR	1.132	0.623	0.251	
	95%-CI	[-0.451; 0.699]	[-1.033; 0.085]	[-2.053; -0.711]	
	p	0.672	0.096	<0.001	

Age 6-12y: GRASP Preference differences between Phases

		pre-contrast	arterial	portal venous	delayed venous
pre-contrast	OR		0.193	0.461	0.363
	95%-CI		[-2.557; -0.737]	[-1.508; -0.041]	[-1.868; -0.159]
	p		<0.001	0.038	0.020
arterial vs	OR	5.191		2.392	1.884
	95%-CI	[0.737; 2.557]		[-0.051; 1.796]	[-0.226; 1.493]
	p	<0.001		0.064	0.149
Portal venous vs	OR	2.170	0.418		0.788
	95%-CI	[0.041; 1.508]	[-1.796; 0.051]		[-1.087; 0.609]
	p	0.038	0.064		0.581
delayed venous	OR	2.755	0.531	1.270	
	95%-CI	[0.159; 1.868]	[-1.493; 0.226]	[-0.609; 1.087]	
	p	0.020	0.149	0.581	

Age >12y: GRASP Preference differences between Phases

		pre-contrast	arterial	portal venous	delayed venous
pre-contrast	OR		0.104	0.257	0.361
	95%-CI		[-3.265; -1.255]	[-2.194; -0.525]	[-1.79; -0.247]
	p		<0.001	0.001	0.010
arterial vs	OR	9.581		2.461	3.461
	95%-CI	[1.255; 3.265]		[-0.143; 1.944]	[0.27; 2.213]
	p	<0.001		0.091	0.012
Portal venous vs	OR	3.893	0.406		1.406
	95%-CI	[0.525; 2.194]	[-1.944; 0.143]		[-0.515; 1.197]
	p	0.001	0.091		0.435
delayed venous	OR	2.769	0.289	0.711	
	95%-CI	[0.247; 1.79]	[-2.213; -0.27]	[-1.196; 0.515]	
	p	0.010	0.012	0.435	
